# Oncology Health Management: From Educational Awareness to System Building

**DOI:** 10.1002/cai2.70061

**Published:** 2026-04-13

**Authors:** Xinyue Bao, Bo Lan, Fei Ma

**Affiliations:** ^1^ Department of Medical Oncology National Cancer Center/National Clinical Research Center for Cancer/Cancer Hospital, Chinese Academy of Medical Sciences and Peking Union Medical College Beijing China

Malignant tumors remain a major threat to human health worldwide. According to the latest global cancer statistics, approximately 19.97 million new cancer cases are diagnosed each year, underscoring the growing burden of cancer worldwide [1]. The disease‐centered multidisciplinary team (MDT) model has substantially improved prognosis and extended survival in many malignancies. Yet longer survival has also made the long‐term consequences of cancer and its treatment more visible. Age‐related comorbidities, pre‐existing conditions, and treatment‐related toxicities can all shape outcomes well beyond tumor control. As a result, the risk of non‐cancer mortality associated with cancer therapy has become increasingly important. In breast cancer, for example, prolonged treatment may be accompanied by cardiovascular events, skeletal complications, secondary malignancies, reproductive impairment, and depression. These problems can seriously compromise quality of life and, in some patients, may even outweigh the mortality risk posed by the tumor itself [2]. Together, these challenges highlight the limits of a treatment model focused primarily on the tumor and point to the need for a broader, patient‐centered approach to cancer care.

Against this background, oncology health management has emerged as a useful framework for rethinking cancer care. In our view, this framework can be understood along two dimensions: space and time. Spatially, it calls for a shift from a disease‐centered MDT model to a patient‐centered interdisciplinary team (IDT) model, with closer integration of fields such as cardio‐oncology, oncofertility, and endo‐oncology [3]. Temporally, it expands the scope of care across the full disease trajectory, from prevention, screening, and early detection to acute treatment, long‐term follow‐up, and rehabilitation. In this sense, oncology health management is not simply an extension of anticancer treatment; it is an effort to address the broader health needs of patients throughout the cancer continuum.

A practical starting point for this model is medical education. Developing a patient‐centered and holistic perspective early in training may help future physicians recognize that good cancer care involves more than tumor response alone. With this goal in mind, our team launched the course “Oncology Health Management” at Peking Union Medical College 3 years ago. The course is organized around two themes: the full life cycle of cancer care and comprehensive interdisciplinary management. It covers prevention and screening, MDT‐based acute treatment, and chronic‐phase follow‐up and rehabilitation, while also introducing areas that are often underemphasized in conventional oncology teaching, including cardio‐oncology, pulmonary oncology, endo‐oncology, and oncofertility. To assess its educational value, we used paired pre‐ and post‐course questionnaires. The results showed improved student understanding of issues beyond direct antitumor therapy, particularly cross‐system complications and long‐term treatment‐related risks. Students demonstrated greater awareness of topics such as early recognition of interstitial pneumonitis associated with targeted or immunotherapy, reproductive impairment caused by anticancer drugs, and the long‐term adverse effects of endocrine therapy. These findings suggest that the course helps broaden students' clinical perspective and encourages a more interdisciplinary view of cancer care.

Education alone, however, is not enough. Broader implementation of oncology health management also requires sustained dialogue within the clinical community. In parallel with our teaching efforts, our team has organized the China Oncology Health Management Congress annually for several consecutive years. The Congress extends beyond the traditional single‐disease format of many oncology meetings and instead emphasizes oncology's intersections with pulmonology, cardiology, neurology, reproductive medicine, and endocrinology. It has consistently promoted the idea of “whole‐process, whole‐person” care, while creating space for discussion of cancer‐related quality of life, patient‐reported outcomes (PROs), and condition‐specific assessment tools such as the NCC‐BC‐A scale [4]. By encouraging clinicians to weigh long‐term quality of life alongside antitumor efficacy, the Congress helps translate the concept of oncology health management into a more practical and measurable clinical agenda.

To support longer‐term development of the field, our team has also undertaken the compilation of a monograph, *Oncology Health Management*, to be published by People's Medical Publishing House. The project brings together specialists from oncology disciplines including medical oncology, surgery, radiation oncology, imaging, pathology, and cancer prevention, as well as experts from cardiology, pulmonology, neurology, nephrology, and psychosomatic medicine. The aim is to convert fragmented clinical experience into a more systematic body of knowledge and to provide trainees and clinicians with an accessible interdisciplinary reference for cancer care across the disease trajectory.

Taken together, these efforts represent one attempt to move oncology health management from educational awareness toward system building. Through a combination of curriculum development, academic exchange, and interdisciplinary writing, we hope to support a more patient‐centered, longitudinal, and integrated model of cancer care. Although this framework is still evolving, it may offer a useful direction for improvinglong‐term outcomes and quality of life in patients with cancer.

## Author Contributions

**Xinyue Bao:** writing – original draft. **Bo Lan:** writing – review and editing, conceptualization (equal), project administration (equal), supervision (equal). **Fei Ma:** conceptualization (equal), project administration (equal), supervision (equal).

## Ethics Statement

The authors have nothing to report.

## Consent

The authors have nothing to report.

## Conflicts of Interest

Professor Fei Ma is the member of the *Cancer Innovation* Editorial Board. To minimize bias, he was excluded from all editorial decision‐making related to the acceptance of this article for publication. The remaining authors declare no conflicts of interest.

## Data Availability

The authors have nothing to report.
